# Age differences in pro-environmental behaviors: Is it about me or future generations?

**DOI:** 10.1007/s10433-026-00918-9

**Published:** 2026-03-18

**Authors:** Zhixuan Lin, Helene H. Fung

**Affiliations:** 1 Department of Psychology, The Chinese University of Hong Kong, Hong Kong, SAR, China; 2 Department of Psychology, University of Zurich, Zurich, Switzerland

**Keywords:** Pro-environmental behaviors, Age differences, Risk perceptions, Social generativity, Socioemotional selectivity theory, Self-transcendence

## Abstract

With climate change and population aging, it has become important to motivate older adults to engage in pro-environmental behaviors. Compared with younger adults, older adults may have lower ecological risk perceptions (perceived personal harm and worry) and higher social generativity (concern for future generations). The former may make them less likely, while the latter may make them more likely, to engage in pro-environmental behaviors. This study examined age-related pro-environmental behaviors when these two factors worked together. A survey was conducted among 235 older (Mage = 70.64, age range 64–93) and 238 younger adults (Mage = 25.91, age range 19–37) in Hong Kong, a subtropical city facing increasing temperatures. Despite no significant age differences in ecological risk perceptions, older adults reported greater recycling, political support, consumption-related pro-environmental behaviors, and higher social generativity compared to younger adults. Younger adults exhibited the same level of pro-environmental behaviors as older adults did at higher levels of social generativity. When asked why they engaged in pro-environmental behaviors, older adults reported more pro-environmental, habitual, and norm-conforming reasons, while younger adults reported more monetary reasons. These findings suggest that although older adults do not perceive higher ecological risks, they may engage in more pro-environmental actions for the sake of future generations. Implications for future studies and practices were discussed.

**Supplementary Information:**

The online version contains supplementary material available at 10.1007/s10433-026-00918-9.

## Introduction

“*I personally don*’*t think climate change is a big issue, but I*’*m still willing to practice the (pro-environmental) behaviors for my grandson*”. *– One older participant from our project.*

Aging population and climate change stand out as critical global challenges in the twenty-first century. There is a significant increase in the global share of individuals aged 65 and over, nearly doubling from 5.5% in 1974 to 10.3% in 2024 (UNFPA, 2024). Meanwhile, the frequency and intensity of extreme hot temperatures are on the rise each year (NASA, 2024). Within this landscape, Hong Kong, a subtropical urban city with high population density, faces both the challenges of aging and climate change. With 19.6% of the population aged 65 years and above (Census and Statistics Department of Hong Kong, 2021) and one of the world’s highest increases in ambient temperature during the past century (United Nations Development Programme, 2022), Hong Kong serves as a prototype to study these converging issues.

Given this context, involving older adults in environmental improvement endeavors becomes important. Previous studies have identified several factors that facilitate pro-environmental behaviors, such as risk perceptions and perceived environmental threats (e.g., Bradley et al. 2020; McCright 2010). Other studies identified generativity as an important factor for pro-environmental behaviors (e.g., Urien & Kilbourne 2011; Wells et al. 2016). These factors can be categorized in two major motivations: self-relevance, which refers to whether individuals perceive the risks and threats of environmental issues and feel responsible for them, and self-transcendence, which involves concerns and readiness to act for future generations, irrespective of whether they feel the harm personally.

Older age has been found to be related to both categories of motivations. On the one hand, older adults were found to have less concern and knowledge about climate change, indicating a lower level of risk perceptions (Pillemer et al. 2022; Sorour et al. 2025). On the other hand, they consistently show a greater concern for the welfare of future generations (for a review, see Villar et al. 2023). The former pathway could lead to fewer, while the latter pathway could lead to more, pro-environmental behaviors among older adults compared to younger adults. Recent studies have started to pay attention to older adults as activists amid climate change (e.g., Ayalon et al. 2022; Smith & Kingston 2021; Wang et al. 2021). But none of the prior studies have examined these two competing forces simultaneously to investigate whether they overall lead to more or fewer pro-environmental behaviors among older relative to younger adults. Moreover, it remains unknown whether these factors contribute to older adults’ pro-environmental behaviors to the same degree as they do for younger adults. The current study investigates age group differences in pro-environmental behaviors, and the roles of ecological risk perception and social generativity in them. Furthermore, reasons for engaging in pro-environmental behaviors and their age differences were explored.

### Relationship between ecological risk perceptions, social generativity, and pro-environmental behaviors

Pro-environmental behaviors are defined as actions by individuals that have a beneficial impact on the environment, such as reuse, recycling, eco-friendly consumption, and household energy conservation. The positive association between ecological risk perceptions and pro-environmental behaviors has been well documented in previous literature (e.g., Bradley et al. 2020; van der Linden 2015). This relationship can be explained in the framework of the theory of planned behaviors (TPB, Ajzen 1991). When applying the theory to pro-environmental behaviors, Bradley et al. (2020) found that risk perceptions was one critical attitude that predicted the intention of pro-environmental behaviors. Although climate change indeed has considerable impacts on human society, it is sometimes difficult for people to detect or experience its harm at an individual level (Van der Linden 2017). Avoidance and denial of climate change issues, representing low ecological risk perceptions, could result in the absence of pro-environmental behaviors (Wullenkord & Reese 2021). Higher risk perceptions, indicating that individuals are concerned about environmental issues or may have been affected by them (van der Linden 2015), lead to more pro-environmental behaviors (Bradley et al. 2020).

Social generativity has been examined as another crucial factor (e.g., Urien & Kilbourne 2011; Wang et al. 2023; Wells et al. 2016). Generativity has two broad dimensions: one that is more agentic, targeting symbolic immortality, and another that is more communal and altruistic, focusing on the concern for and the desire to contribute to future generations (McAdams & de St. Aubin, 1992). Social generativity embodies the latter (Morselli & Passini 2015). Individuals with higher social generativity have desires to live in a way that their actions have a positive impact beyond their own life (Kotre 1984). As climate issues intensify, even though people sometimes may not perceive their threats at present, they are aware that future generations will be more impacted (Hansen et al. 2013). In that case, social generativity concerns work as a self-transcendence motivation that promote pro-environmental behaviors (Giménez García-Conde et al. 2016). This has been supported by empirical evidence showing positive relationships between social generativity and pro-environmental intentions and behaviors, such as purchasing eco-friendly products (Wang et al. 2023) and water and energy saving (Wells et al. 2016).

Despite both risk perceptions and social generativity having been examined in previous studies, it remains unclear how they may influence age-related pro-environmental behaviors when they co-exist. Neither do we know whether they correlate with older and younger adults’ pro-environmental behaviors differently.

### Age differences in pro-environmental behaviors

There have been contradictory findings regarding age differences in pro-environmental attitudes and behaviors. Recent studies have showed that compared with older adults, younger adults are more conscious of environmental issues, which is supposed to lead to increased pro-environmental behaviors. For example, younger adults display more positive attitudes toward sustainable behaviors (Wiernik et al. 2013), as well as higher levels of eco-anxiety (Ágoston et al. 2024). However, this does not appear to be the case regarding behaviors, or at least self-reported behaviors. A recent study across 31 countries has revealed a positive correlation between age and self-reported pro-environmental behaviors at both the individual level (i.e., older people participate in more pro-environmental behaviors than younger adults) and the national level (i.e., countries with a higher proportion of older adults score higher on pro-environmental behaviors; Wang et al. 2021). A meta-analytic study showed that older adults reported more activities that aim to avoid environmental harm and conserve materials, such as reusing and recycling, than were younger adults (Wiernik et al. 2013).

Why older adults engage in more pro-environmental behaviors even though they have lower environmental-related attitudes compared to younger adults? One potential explanation may lie in the distinctions between concern for oneself and for others/future generations. Psychological distance regarding climate change affects pro-environmental behaviors (Maiella et al. 2020). van der Linden (2015) suggested that the impact of climate change was often perceived as more psychologically distant, both spatially and temporally, for oneself than for future generations. As older adults tend to perceive future time as more limited (Lang & Carstensen 2002), they may value the two concerns differently. Older adults may be less likely to view themselves as victims of climate change, potentially leading to lower risk perceptions for themselves. Meanwhile, a limited future time perspective may also result in higher other-focused concerns and self-transcendent values (Brandtstädter et al. 2010). How these two forces counteract with each other and relate to older adults’ pro-environmental behaviors remains unknown.

### Age differences in ecological risk perceptions and social generativity

Older adults may have lower ecological risk perceptions compared to younger adults. First, a “positivity bias” in attention and memory is observed among older adults (Mather & Carstensen 2005), suggesting that they pay more attention to positive information relative to negative one compared to younger adults (Isaacowitz et al. 2006). This could be rooted in older adults’ increased motivation to optimize their emotions when perceiving future time as limited (Carstensen et al. 1999). In this vein, they may be more likely to look away from climate change risk information that induces negative emotions such as eco-anxiety (Ágoston et al. 2024). Second, as mentioned above, sometimes it is hard for people to experience the harm of climate change at an individual level (Van der Linden 2017), and the impact of climate change may be more salient in the future compared to now (Hansen et al. 2013). Under a limited future time perspective (Carstensen et al. 1999), older adults are less likely to perceive themselves as potential victims of climate change. Consequently, they tend to have lower risk perception compared to younger adults. Previous studies support this by finding that younger age is associated with higher eco-anxiety and worry (Ágoston et al. 2024; Boluda-Verdú et al. 2022). As risk perceptions serve as a prerequisite for pro-environmental behaviors, younger adults are more likely to engage in pro-environmental behaviors than are older adults.

Nevertheless, despite less concerns for their own sake, older adults could be more worried about and willing to contribute for the sake of generations that have yet to come. Erikson depicted generativity as the seventh of eight developmental stages, which is the developmental task for middle age (Erikson & Erikson 1998). Other researchers have found that generativity continues into older adulthood linearly (Schoklitsch & Baumann 2012). Age has been found to be positively associated with self-transcendent values that aim to extend meaning beyond the present self (Brandtstädter et al. 2010). Social generativity is a typical self-transcendent value that is closely related to the altruistic and transmission aspect of generativity, namely, the aspect that focuses on responsibility for future generation (Morselli & Passini 2015). Social generativity has been found to positively correlate with chronological age (Guiot et al. 2019) and a limited future time perspective (Kooij & Van De Voorde, 2011), indicating potentially more pro-environmental behaviors among older adults compared to younger adults.

### Other reasons for pro-environmental behaviors

Individuals can engage in pro-environmental behaviors without the intention to benefit the environment. Many environmental behaviors are confounded with saving behaviors, such as energy conservation, reuse, and recycling (Gkargkavouzi et al. 2019; Guiot et al. 2019; Wells et al. 2016). Hong Kong’s electricity bills are considered expensive compared to the global average and residents need to pay for plastic bags in groceries. In that regard, they may save energy in their households and use recycled bags for monetary reasons. According to the TPB, personal norm and social norm are also important reasons for engaging in pro-environmental behaviors (Ateş, 2020). The current cohort of older adults in Hong Kong may be more likely to live a frugal lifestyle due to lower economic development levels during their childhood compared to the current younger generations. This may translate into habits, even after their economic status has significantly improved (Whitebread & Bingham 2013). Moreover, older adults endorse social norms more strongly than do younger adults (Fung 2013). In a society with a phenomenon of acting green, older adults may simply follow this to be in line with others.

## Current study

The current study examined age group differences in ecological risk perceptions, social generativity concerns, pro-environmental behaviors (self-reported), and their relationships. We proposed that both ecological risk perceptions (H1) and social generativity concerns (H2) positively correlated with self-reported pro-environmental behaviors. Older adults may simultaneously report lower levels of ecological risk perceptions (H3) and higher levels of social generativity concerns (H4) compared to younger adults, which may be associated with different directions of age differences in pro-environmental behaviors. Given that, we did not have a hypothesis regarding whether older or younger adults would report more pro-environmental behaviors and kept it as an open research question (RQ1). We further explored whether age groups would moderate the relationships between ecological risk perceptions, social generativity concerns, and pro-environmental behaviors, to answer the question regarding whether these factors correlated with older and younger adults’ pro-environmental behaviors differently (RQ2). We also explored the extent to which older and younger adults’ self-reported pro-environmental behaviors stemmed from the following four reasons: pro-environmental (to benefit environment), habitual, norm-conforming, and monetary (to save money) reasons (RQ3).

### Participants

Our data were collected as part of a broader project (the “Extreme Weather” project) in Hong Kong. The project broadly aimed at examining the impact of temperature on older adults’ well-beiong. The pre-registration and materials of the project can be found at the Open Science Framework (OSF: 10.17605/OSF.IO/57VSG). The project was approved by the corresponding author’s affiliation (Reference No. SBRE‐22‐0020).

Participants were recruited through mass email and advertisement on a local newspaper. Community-dwelling older (aged 60 years and above) and younger (aged between 18 and 40 years) who could read Cantonese Chinese were recruited. The data collection was conducted in two years, 2023 and 2024, in person. Student assistants visited participants in their homes to collect measures included in this study with tablets. The two-year data were combined for analysis due to invariance in demographic distribution and results (see Table 1). To test this, the year was included as a covariate as well as a moderating factor. It did not moderate the observed relationships (see Supplemental Materials Table 4). Older participants’ self-reported health and cognitive ability suggest that they had no functional dependence (see Table 1). All participant are urban residents, representing the population of Hong Kong, where almost 100% of residents live in urban area (Li & Bou-Zeid 2013). Further information on the representation of this sample can be found in Supplementary Materials.

**Table 1:** Demographic distribution of younger and older adults

Year	2023		2024	
No., % or Mean (SD)	YA	OA	YA	OA
No. of participants	118	130	120	105
Age	25.79 (4.82)	70.60 (5.16)	26.04 (4.90)	71.70 (5.16)
Age range	19–37	65–85	20–37	64–93
Gender (female %)	67.2	61.1	63.3	50.5
Married (%)	8.4	61.1	11.5	68.7
*Income level (HKD, %)*				
$0–3,000	3.4	16	0.8	18.1
$3,001–8,500	2.5	24.4	5.0	13.3
$8,501–14,000	3.4	15.3	6.7	11.4
$14,001–20,000	10.9	9.2	15.0	10.5
$20,001–30,000	12.6	8.4	20.0	11.4
$30,001–60,000	40.3	17.6	29.2	16.2
$60,001–100,000	21	3.8	7.5	11.4
$ > 100,000	4.2	3.1	8.3	1.9
Subjective SES	4.97 (1.33)	4.42 (1.70)	4.85 (1.30)	5.35 (1.74)
*Educational level (%)*				
Primary school or below	0	8.1	0	9.7
Junior (high) school	1.0	60.8	2.8	59.1
Some college	0	9.5	0	7.5
Bachelor's degree	63.5	14.9	76.1	15.1
Master’s degree and above	35.6	6.8	21.1	8.6
Subjective health	3.02 (0.92)	2.69 (0.84)	2.94 (0.88)	2.98 (0.86)
Cognitive health	NA	26.94 (3.24)	NA	27.85 (2.24)

YA = younger adults, OA = older adults. SES = socioeconomic status

A total number of 240 older (*M*_age_ = 69.54, SD_age_ = 5.67, age range = 64–93, 59.2% females) and 238 younger adults (*M*_age_ = 25.41, SD_age_ = 6.52, age range = 19–37, 67.5% females) participated in the study. Five older participants were excluded due to dropouts and missing responses. Older participants were reimbursed with HKD 600, and younger adults were reimbursed with HKD 200. The older participants were reimbursed more because they provided additional psycho-physio data, which was not included in the current analysis. Post-hoc sensitivity tests show that the total sample size (*N* = 473) provides 80% statistical power to detect a minimum effect size with six predictors of *f*^2^ = 0.029 at *α* = 0.05 level (G*Power, Faul et al. 2007), and provides greater than 90% power to detect the observed interaction using “InteractionPoweR” in R CRAN (Baranger et al. 2023).

### Measures

*Pro-environmental behavior* was self-reported by a multi-dimensional scale (Gkargkavouzi et al. 2019). The original scale has 22 items. We deleted 8 items as citizens in Hong Kong seldom carry out those behaviors. The final scale consisted of 14 items, encompassing six sub-dimensions: civic actions (3 items, e.g., “*I participate in community events/workshops which focus on environmental awareness*”.), policy support (2 items, e.g., “*I systematically write letters to politicians or candidates for environmental issues*”.), recycling (3 items, e.g., “*I recycle paper, glass and aluminium packages*”.), transportation (1 item, “*I ride a bicycle or take public transportation instead of driving cars*”.), household settings (2 items, e.g., “*I wait until I have a full load before doing my laundry*”.), and consumption (2 items, e.g., “*I use my own bags instead of plastic ones when I go shopping*”.). Each item was evaluated on a 5-point Likert scale ranging from 1 (never) to 5 (always). Mean scores were calculated for each sub-dimension as well as for all items collectively. The scale’s Cronbach’s alpha in this sample was 0.741. The final scale yielded a good model fit of both single-dimension model (CFI = 0.959, TLI = 0.911, SRMR = 0.036) and six-dimension model (CFI = 0.931, TLI = 0.900, SRMR = 0.043). Metric invariance was achieved across two age groups, which allowed us to compare the associations between variables. We used sub-dimensions to examine means differences between age groups. Statistics examining the associations between sub-dimensions and other variables can be found in Tables 1, 2 and 6, and group invariance tests can be found in Table 7 of Supplementary Materials.

*Reasons for pro-environmental behavior* were assessed for each sub-dimension of pro-environmental behavior scale with a sub-sample of 120 younger and 105 older adults in Wave 2024. After a participant has reported a pro-environmental behavior, four reasons were assessed: 1. Pro-environmental reason (“*I do this because it benefits the environment*”), 2. Habitual reason (“*…because it is my habit*”), 3. Norm conformity (“*…because other people are doing so*”), and 4. Saving money (“*…to save money*”). Participants responded on a 5-point Likert scale ranging from 1 (does not align with my reason at all) to 5 (align very much). Mean scores across all sub-dimensions of pro-environmental behaviors are calculated for each reason.

*Ecological risk perception* was measured by climate change risk perceptions scale (CCRPs, Stevenson et al. 2015). The scale consisted of four questions. The first two questions assessed to what extent participants were worried about and were harmed by climate change personally. The third question pertained to the sense of urgency (i.e., perception of when climate change would begin to harm people in their city). The fourth question examined participants’ perception of the extent to which climate change would impact future generations. This item may not tap into self-relevant concern but rather self-transcendent concern. Except for the third question that could be rated from 0 (the present) to 100 (100 years or later), the other questions were evaluated on a 4-point Likert scale ranging from 1 (not at all) to 4 (extremely). Recognizing that the four items may capture different aspects of ecological risk perception, we examined them in analysis separately.

*Social generativity* was measured by social generativity scale (SGS, Morselli & Passini 2015). The scale consisted of six items (e.g., “I think that I am responsible for ensuring a state of well-being for future generations”). Each item was evaluated on a 7-Likert point scale ranging from 1 (totally disagree) to 7 (totally agree). Mean scores were calculated for all items collectively. The scale's Cronbach’s alpha in this sample was 0.917.

Participants reported their gender, age, education level, family income level (see Table 1), subjective health, subjective socioeconomic status (SES), and completed the Hong Kong version of Montreal Cognitive Assessment (HK-MoCA, Yeung et al. 2014). As no participant scored below the cut-off point of mild cognitive impairment and dementia (21/22), no participant was dropped from the study based on cognitive ability. Subjective health was measured by one item regarding their overall evaluation of their health status, scaling from 1 to 5. Subjective SES was measured using the MacArthur scale (Adler et al. 2000), which use a 10-rung social ladder to represent how individuals rank themselves from the bottom to the top of society.

## Results

### Descriptive statistics, correlations, and age differences for the key variables

Descriptive statistics, age differences, and the correlational matrix for the key variables are summarized in Tables 2 and 3. Older adults reported greater social generativity and more recycling, political support, and consumption-related pro-environmental behaviors than did younger adults. However, younger and older adults were not significantly different in any of the four items related to ecological risk perception. The correlation between social generativity and pro-environmental behaviors yielded a large effect among younger adults (*r* = 0.49) and a small-to-medium effect among older adults (*r* = 0.23; Cohen 1988). Risk perception correlated with pro-environmental behaviors to a similar degree among older and younger adults (*r* ranges from 0.14 to 0.30), with the exception of a nonsignificant correlation between urgency perception and pro-environmental behaviors among older adults.

**Table 2:** Age group differences in sub-dimensions of pro-environmental behaviors

	Age Group	Mean	SD	*t* (471)	*p*	Cohen’s* d*
Civic actions	YA	1.45	0.61	− 0.739	.460	− .068
	OA	1.50	0.61			
Political support	YA	1.16	0.45	− 4.631	< .001	− .426
	OA	1.42	0.74			
Recycling	YA	2.97	0.83	− 7.938	< .001	− .730
	OA	3.92	0.94			
Transportation	YA	3.99	1.17	1.795	.073	.165
	OA	3.77	1.25			
House settings	YA	3.99	0.79	− .947	.344	− .087
	OA	4.05	0.81			
Consumption	YA	3.28	0.81	− 8.463	< .001	− .778
	OA	3.87	0.71			

YA = younger adults, OA = older adults

**Table 3:** Correlations between the key variables

	YA	OA								
	M (SD)	M (SD)	t (471)	Cohen’s *d*	1	2	3	4	5	6
1. CCRPs_Worry	2.57(0.79)	2.52(0.97)	0.62	.06		.70^***^	− .05	.55^***^	.35^*^	.31^**^
						[.63, .76]	[− .18, .08]	[.45, .63]	[.23, .46]	[.19, .42]
2. CCRPs_Harm	2.58(0.70)	2.52(0.83)	0.89	.08	.59^***^		− .03	.55^***^	.32^**^	.24^**^
					[.50, .67]		[− .16, .10]	[.45, .63]	[.20, .43]	[.12, .36]
3. CCRPs_Urgency	26.01(26.02)	22.87(26.93)	1.29	.12	− .21^*^	− .25^*^		− .11	− .00	.02
					[− .33, − .09]	[− .36, − .12]		[− .23, .02]	[− .24, .10]	[− .11, .14]
4. CCRPs_Future	3.25(0.68)	3.22(0.75)	0.47	.04	.45^***^	.54^***^	− .30^**^		.26^**^	.18^**^
					[.33, .54]	[.41, .62]	[− .41, − .18]		[.14, .38]	[.05, .30]
5. SGS	5.06(1.08)	5.77(0.90)	− 7.76^***^	− .71	.40^***^	.35^***^	− .12	.22^**^		.23^**^
					[.29, .50]	[.24, .46]	[− .24, .01]	[.10, .34]		[.11, .35]
6. PEB	2.67(0.47)	2.92(0.52)	− 5.49^***^	− .51	.30^***^	.21^***^	− .14^*^	.19^**^	.49^***^	
					[.18, .41]	[.08, .33]	[− .27, − .02]	[.06, .31]	[.39, .58]	

The lower triangle shows the results for younger adults, and the upper triangle shows the results for older adults. CCRPs = climate change risk perceptions. CCRPs_Worry = “How worried are you about climate change?”. CCRPs_Harm = “How much do you think climate change harms you personally?”. CCRPs_Urgency = “When do you think climate change will start to harm people in your city?”. The mean and SD of CCRPs_Urgency are the original scores. It was reversely coded for correlation. CCRPs_Future = “How much do you think climate change will harm future generations?”. SGS = social generativity scale. PEB = pro-environmental behavior. ^***^ means *p* < .001, ^**^ means *p* < .01, ^*^ means *p* < .05, ^†^ means *p* < .10

Age differences in reasons for pro-environmental behaviors are shown in Supplementary Materials Table 1. Older adults scored higher than younger adults on environmental, habitual, and norm-conforming reasons, while younger adults scored higher on the reason of saving money.

### Multiple regressions

**Fig. 1:**
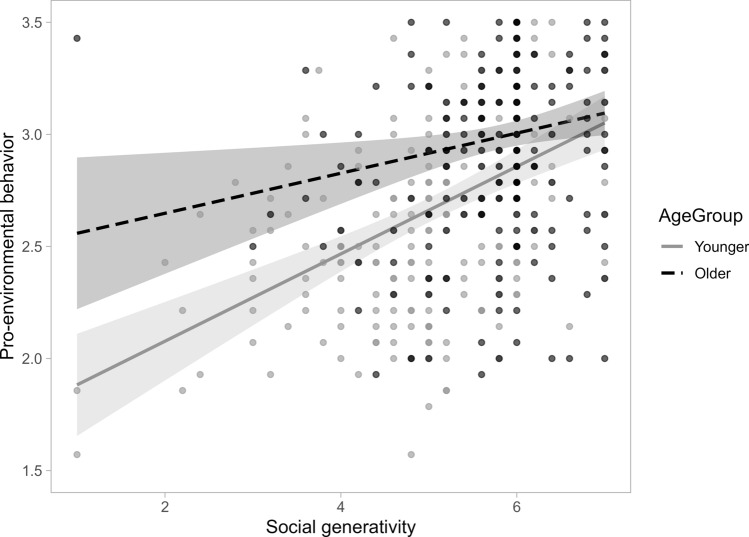
Interaction between age group and social generativity on pro-environmental behavior. Note: The shadow represents the 95% confidence interval

The results of multiple regressions were shown in Table 4. First, we regressed pro-environmental behavior on age group (Model 1). Older adults reported more pro-environmental behavior compared to younger adults. Next, we regressed pro-environmental behavior on age group, the four items of ecological risk perception, and social generativity (Model 2). Eco-worry and social generativity positively predicted pro-environmental behavior. Social generativity explained approximately 10% of the variance, and risk perceptions explained lower than 4% of the variance in pro-environmental behaviors. Lastly, we added in the interaction terms between age group and each item of risk perception and social generativity (Model 3). Only social generativity significantly interacted with age group. Social generativity positively correlated with pro-environmental behavior to a greater extent among younger adults (*beta* = 0.19, 95% CI [0.14, 0.25], *t* = 7.01, *p* < 0.001) than among older adults (*beta* = 0.09, 95% CI [0.02, 0.16], *t* = 2.53, *p* = 0.01). As mentioned above, younger adults reported a lower level of pro-environmental behaviors than did older adults. Yet, younger adults with higher social generativity concern reported similar levels of pro-environmental behaviors as older adults (Fig. 1). The above results remained unchanged even after controlling for subjective health, subjective SES, and income (see Supplementary Materials Table 3). Regression models with sub-dimensions of pro-environmental behaviors as DVs can be found in Supplementary Materials Table 7.

**Table 4:** Standardized regression coefficients (*beta*) and 95% confidence intervals (*CIs*) for hierarchical regression models predicting pro-environmental behaviors

	Model 1				Model 2				Model 3			
Predictors	beta	95% CI	p	Partial R^2^	beta	95% CI	p	Partial R^2^	beta	95% CI	p	Partial R^2^
AG	.31	[.23, .40]	** < .001**	.098	.20	[.12, .29]	** < .001**	.047	.21	[.13, .30]	** < .001**	.005
CCRPs_Worry					.16	[.05, .27]	**.004**	.017	.15	[.04, .26]	**.006**	.005
CCRPs_Harm					.01	[− .10, .12]	.850	< .001	− .01	[− .12, .10]	.925	.001
CCRPs_Urgency					.01	[− .07, .08]	.901	< .001	.00	[− .08, .08]	.979	.002
CCRPs_Future					.05	[− .04, .15]	.278	.003	.05	[− .04, .15]	.294	.001
SGS					.32	[.23, .41]	** < 0.001**	.096	.30	[.21, .39]	** < .001**	.096
CCRPs_Worry × AG									.03	[− .08, .14]	.600	.001
CCRPs_Harm × AG									.06	[− .05, .17]	.281	.003
CCRPs_Urgency × AG									.06	[− .02, .14]	.121	.005
CCRPs_Future × AG									.00	[− .10, .10]	.980	< .001
SGS × AG									− .11	[− .20, − .02]	**.019**	.012
Adjusted R^2^/R^2^ change	.096				.263/.174***				.269/.013*			

^***^ means *p* < .001, ^**^ means *p* < .01, ^*^ means *p* < .05. CCRPs = climate change risk perceptions. SGS = social generativity concerns. AG = age groups. *Partial R*^2^ represents the unique variance explained by each predictor

Reasons for pro-environmental behaviors were measured after pro-environmental behaviors, potentially serving as a post-hoc rationalizaiton. Thus, we do not think it is appropriate to regress pro-environmental behaviors on reasons. Yet, such analysis is available in Supplementary Materials Table 5.

## Discussion

The current study examined age group differences in pro-environmental behaviors and the roles of ecological risk perceptions and social generativity in them. Findings revealed that older adults reported more recycling, political support, and consumption-related pro-environmental behaviors and social generativity than younger adults did. Social generativity positively predicted pro-environmental behaviors, but to varying degrees among older and younger adults. Ecological risk perceptions did not differ between younger and older adults. Despite significant zero-order correlations with pro-environmental behaviors, only eco-worry, but not any other aspect of ecological risk perception, significantly predicted pro-environmental behaviors after controlling for the effects of age groups and social generativity.

These findings suggest that older adults reported more pro-environmental behaviors (recycling, political support, and consumption) than did younger adults without perceiving climate change as more severe and pressing. As climate change is often characterized as a psychologically distant risk that is more likely to happen in the “future” and to “other” people (Spence et al. 2012; van der Linden 2015), concerns for future generations may be more predictive of pro-environmental behaviors than perceived harm for oneself. In our samples, both older and younger adults perceive climate change as significantly impacting local citizens 20 years from now. However, this perception may have different meanings for the two age groups. After 20 years, younger adults will be around 40, while older adults will be around 90 or may have already passed away. This may be the reason why such urgency perception failed to predict older adults' pro-environmental behaviors. As older age is associated with increased self-transcendent goals over instrumental or self-interest goals (Brandtstädter et al. 2010), it is plausible that the effects of self-transcendent goals override the effects of self-relevant goals (e.g., perceived risk on oneself), resulting in higher pro-environmental behaviors in older age.

We further investigated whether ecological risk perceptions and social generativity correlated with older and younger adults’ pro-environmental behaviors differently. Risk perceptions positively correlated with pro-environmental behaviors among both younger and older adults to a similar degree. Social generativity correlated with pro-environmental behaviors among younger to a greater degree than among older adults. Even though older adults reported a higher level of pro-environmental behaviors than did younger adults, the gap was narrowed with higher levels of social generativity. This could potentially mean that social generativity is one mechanism for explaining the observed age group differences in pro-environmental behaviors. Previous research has found that older adults have higher generativity concerns than do younger adults (McAdams et al. 1993), and generativity concerns are related to more pro-environmental behaviors (Urien & Kilbourne 2011; Wang et al. 2023). Our findings link these two literatures together.

For reasons for pro-environmental behaviors, older adults scored higher than younger adults on pro-environmental, habitual, and norm-conforming reasons for pro-environmental behaviors. This provides evidence that older adults may indeed aim to benefit the environment when they self-report engaging in pro-environmental behaviors, not merely to adhere to personal or social norms. TPB (Ajzen 1991) suggests that personal norm, generated from social norm, is an important factor for pro-environmental behaviors. Older adults are more likely to internalize social norms compared to younger adults (Fung 2013), which explains why they also scored higher on habitual and norm-conforming reasons than younger adults. This could be particularly true for the current cohort of older adults in Hong Kong, who grew up in a historical period when society was not as economically developed, and reusing and recycling were their norms of living. Younger adults, on the contrary, are more likely to engage in pro-environmental behaviors to save money, which could be viewed as a more instrumental reason. These findings are in line with the speculation of SST (Carstensen et al. 1999) and self-transcendence theory (Brandtstädter et al. 2010), which propose that material (future-oriented) goals decline with age while transcendent (emotionally meaningful) goals increase.

### Limitations, implications, and future directions

We acknowledge some limitations of the current study. First, this study is cross-sectional, and we did not directly examine potential mechanisms such as future time perspective. It is uncertain whether the observed differences are age-driven changes or cohort differences, which could be clarified in future longitudinal studies. Future time perspective is theorized as one potential mechanism for age-related changes in social generativity (Kooij & Van De Voorde, 2011). Future experimental studies can manipulate individuals’ future time perspectives (e.g., Fung et al. 2020) to examine whether it induces changes in pro-environmental behaviors. Future experimental studies can also manipulate individuals’ eco-anxiety (O’Connor et al. 1999) and generativity concerns (Moieni et al. 2021) to see if they result in divergent levels of pro-environmental behaviors.

Second, we should be cautious regarding generalization of the conclusions. In terms of region and cultural characteristics, Hong Kong is a subtropical city with almost 100% of its population residing in urban areas. People there face more heat stress than other climate issues (e.g., disastrous events). Whether our findings could be generalized to regions facing different climate issues is worth investigating. Hong Kong is characterized by its mixed cultural features, scoring median on the cultural tightness and looseness (Gelfand et al. 2011). It remains unknown whether individuals from cultures higher in tightness may engage in pro-environmental behaviors due to adherence to personal and social norms to a greater extent. Cultures that differ in long-term versus short-term orientation (Hofstede & Minkov 2010) may also show differences in social generativity and eco-urgency perception, and thus may consequently have different levels of pro-environmental behaviors. In terms of types of pro-environmental behaviors, we removed items that are not applicable to Hong Kong context from the original pro-environmental scale (e.g., reforestation). These behaviors can be explored in other cities or by qualitative studies focusing on a small group of people who practice them. Age differences were observed in some pro-environmental behaviors (recycling, political support, and consumption) but not in others. Qualitative methods can also be used to explore whether older and younger adults exhibit different profiles of pro-environmental behaviors.

Third, we acknowledge the modest level of variance explained by the predictors and the risk of using single items. Risk perceptions were examined as separate single items, which carries the risk of measurement error. It should be examined with a validated scale in future studies. In addition to perception, other ecological attitudes should be examined (Kaiser et al. 1999). More nuanced attitudes should be incorporated, such as accountability (i.e., is it human to be blamed for climate change; Wu et al. 2019) and malleability (i.e., can human effort mitigate climate change; Miller et al. 2022) beliefs. Although we asked for reasons behind pro-environmental behaviors, we are still uncertain whether the observed age differences in these behaviors were driven by habitual or instrumental purposes. Future studies can use experimental designs to examine how participants respond when pro-environmental behaviors require changes in habits or incur higher costs.

Lastly, all the measures in this study were self-reported, and the pro-environmental behaviors scale was not specifically developed for older adults. Participants may overestimate their frequency of pro-environmental behaviors as those behaviors are socially desirable (Félonneau & Becker 2008). More objective measures, such as peer evaluations, energy expense bills, laboratory-based pro-environmental behavior task (Lange et al. 2018) and experience sampling methods with photovoice techniques, can be utilized in future studies to reduced social desirability and retrospective biases. Future studies can incorporate both objective and self-reported measures to validate the ecological validity of the pro-environmental behaviors scale. Moreover, although the overall score satisfied metric (factor loadings) invariance between older and younger groups, metric invariance is only partially held for the six-sub-dimension model, with the two groups loading differently on civic activities. This calls for the development and validation of pro-environmental scale for older adults.

Despite the limitations, findings from this study provide important implications for promoting pro-environmental behaviors among older and younger adults. Different promotion strategies can be utilized to encourage older and younger adults’ pro-environmental behaviors. For both age groups, making them aware of the immediate threats of climate change and relate themselves to climate change issues (i.e., eco-worry) can be important and efficient. Older adults are especially vulnerable in terms of physical and psychological health amid extremely hot weather, but they seem to underestimate the risks of it (Leung et al. 2025; Lin et al. 2023). Given that they have already been high on concerns for others and future generations, education and awareness campaigns aimed at raising the risk perceptions on themselves may further facilitate their motivations for pro-environmental actions. For younger adults, emphasizing societal impacts and arousing their care for future generations could be particularly powerful, given that they are lower on this aspect compared to older adults. Moreover, offering opportunities for intergenerational dialogue could be important. Non-governmental organizations (NGOs) could provide platforms to facilitate older adults’ participation in environmental efforts with younger generations and encourage them to share experiences with each other. Intergenerational dialogue has been found to be effective in pro-environmental values transmission among parents and children (Kong & Jia 2023) and can be extended to older and younger generations. This can inspire collective action and a sense of shared responsibility, thus increasing pro-environmental efforts among both parties.

## Supplementary Information

Below is the link to the electronic supplementary material.

Supplementary file1 (DOCX 166 KB)

## Data Availability

Data, syntax, and questionnaires of the study are available on OSF (https://osf.io/xr2bw/?view_only=fe3c07161e9e4123924cde645b5f2f41).
