# Supplementary material for: Age differences in pro-environmental behaviors: Is it about me or future generations?
Source: Eur J Ageing. 2026 Mar 18;23(1):22. doi: 10.1007/s10433-026-00918-9 (PMC13121656; doi:10.1007/s10433-026-00918-9)
Supplement: Supplementary file 1 — Supplementary file1 (DOCX 166 KB) [file 10433_2026_918_MOESM1_ESM.docx]

Supplementary materials for

Age differences in pro-environmental behaviors: Is it about me or future generations?

Part 1 Sample description

The household income level and educational level of our sample are comparable to the general levels of the corresponding generations in Hong Kong. In terms of education, for younger generation, the enrollment rate of in higher education was above 70% in 2015 and keeps growing (UNESCO, 2019). For older generation, approximately 12% of the population aged 55+ years possess a post-secondary degree (Census and Statistics Department of Hong Kong SAR Government, 2021). In terms of household income, the median monthly household income of Hong Kong is HKD 30,000 (Census and Statistics Department of Hong Kong, 2024). The older sample of this study scored lower on household income compared to the younger sample and the median level of Hong Kong, likely because they have retired and their pension per month may not be as high as their salary before retirement. However, this does not necessarily imply that the older sample has a lower socioeconomic status compared to the younger sample, as older adults usually have higher savings than younger adults. The older participants scored slightly higher than younger participants on subjective socioeconomic status, measured by MacArthur’s ten level ladder (Adler et al., 2000).

Reference

Adler, N. E., Epel, E. S., Castellazzo, G., & Ickovics, J. R. (2000). Relationship of subjective and objective social status with psychological and physiological functioning: Preliminary data in healthy, White women. *Health Psychology, 19*(6), 586. https://doi.org/10.1037/0278-6133.19.6.586

Census and Statistics Department of Hong Kong SAR Government. 2021. Thematic Household Survey Report. Retrieved October 16, 2024, from https://www.census2021.gov.hk/en/publications.html

Census and Statistics Department of Hong Kong SAR Government. 2024. Population and Households. Retrieved October 16, 2024, from https://www.censtatd.gov.hk/en/scode500.html

United Nations Educational Scientific, and Cultural Organization (UNESCO). (2019). UNESCO Institute for Statistics. “UIS.Stat”. Retrieved October 16, 2024, from <http://data.uis.unesco.org/>

Table 1 Age differences (independent sample t test) in reasons for pro-environmental behaviors

|  | Age Group | Mean | SD | *t* (223) | *p* | Cohen’s *d* |
| --- | --- | --- | --- | --- | --- | --- |
| R_Environment | YA | 3.68 | 0.81 | -5.143 | <.001 | -.687 |
|  | OA | 4.18 | 0.61 |  |  |  |
| R_Habit | YA | 3.92 | 0.58 | -4.212 | <.001 | -.563 |
|  | OA | 4.22 | 0.50 |  |  |  |
| R_Norm | YA | 2.78 | 0.90 | -3.460 | <.001 | .259 |
|  | OA | 3.20 | 0.89 |  |  |  |
| R_Money | YA | 3.66 | 0.75 | 1.941 | .027 | -.462 |
|  | OA | 3.43 | 0.97 |  |  |  |

Note: YA = younger adults, OA = older adults. Reasons for engaging in behaviors: R_Environment = pro-environmental reason, R_Habit = habitual reason, R_Norm = norm conformity, R_Money = saving money

Table 2 Correlations between key variables among younger and older adults using sub-dimensions of pro-environmental behaviors

| Variable | *M* | *SD* | 1 | 2 | 3 | 4 | 5 | 6 | 7 | 8 | 9 | 10 | 11 | 12 |
| --- | --- | --- | --- | --- | --- | --- | --- | --- | --- | --- | --- | --- | --- | --- |
| 1. Age_group |  |  |  |  |  |  |  |  |  |  |  |  |  |  |
|  |  |  |  |  |  |  |  |  |  |  |  |  |  |  |
| 2. RPs_Worry | 2.55 | 0.88 | -.03 |  |  |  |  |  |  |  |  |  |  |  |
|  |  |  | [-.12, .06] |  |  |  |  |  |  |  |  |  |  |  |
| 3. RPs_Harm | 2.56 | 0.76 | -.04 | .65** |  |  |  |  |  |  |  |  |  |  |
|  |  |  | [-.13, .05] | [.60, .70] |  |  |  |  |  |  |  |  |  |  |
| 4. RPs_Urgency | 24.40 | 26.50 | -.06 | -.12** | -.12** |  |  |  |  |  |  |  |  |  |
|  |  |  | [-.15, .03] | [-.21, -.03] | [-.21, -.03] |  |  |  |  |  |  |  |  |  |
| 5. RPs_Future | 3.25 | 0.71 | -.01 | .51** | .54** | -.19** |  |  |  |  |  |  |  |  |
|  |  |  | [-.10, .08] | [.43, .57] | [.47, .60] | [-.28, -.10] |  |  |  |  |  |  |  |  |
| 6. SGS | 5.43 | 1.04 | .35** | .34** | .28** | -.07 | .20** |  |  |  |  |  |  |  |
|  |  |  | [.27, .43] | [.25, .41] | [.20, .37] | [-.16, .02] | [.11, .28] |  |  |  |  |  |  |  |
| 7. Recycling | 3.30 | 0.94 | .34** | .17** | .12** | -.02 | .17** | .32** |  |  |  |  |  |  |
|  |  |  | [.26, .42] | [.08, .26] | [.03, .21] | [-.11, .07] | [.08, .25] | [.24, .40] |  |  |  |  |  |  |
| 8. Household | 4.02 | 0.80 | .04 | .17** | .15** | -.07 | .11* | .19** | .23** |  |  |  |  |  |
|  |  |  | [-.05, .13] | [.08, .26] | [.06, .24] | [-.16, .02] | [.02, .20] | [.10, .27] | [.14, .31] |  |  |  |  |  |
| 9. Consumption | 3.58 | 0.82 | .36** | .20** | .16** | -.08 | .13** | .43** | .43** | .29** |  |  |  |  |
|  |  |  | [.28, .44] | [.11, .28] | [.07, .25] | [-.17, .01] | [.04, .21] | [.36, .50] | [.35, .50] | [.21, .37] |  |  |  |  |
| 10. Transport | 3.89 | 1.22 | -.08 | .12** | .10* | -.06 | .10* | .10* | .13** | .28** | .11* |  |  |  |
|  |  |  | [-.17, .01] | [.03, .21] | [.01, .19] | [-.15, .03] | [.01, .19] | [.01, .19] | [.04, .21] | [.19, .36] | [.02, .20] |  |  |  |
| 11. Civic | 1.48 | 0.61 | .03 | .24** | .16** | -.00 | .13** | .27** | .32** | .14** | .27** | .12** |  |  |
|  |  |  | [-.06, .12] | [.15, .32] | [.07, .24] | [-.09, .09] | [.04, .21] | [.18, .35] | [.23, .40] | [.05, .23] | [.18, .35] | [.03, .21] |  |  |
| 12. Political | 1.30 | 0.63 | .21** | .16** | .12** | .02 | .05 | .24** | .16** | -.01 | .24** | -.02 | .46** |  |
|  |  |  | [.12, .29] | [.07, .24] | [.03, .21] | [-.07, .11] | [-.04, .14] | [.16, .33] | [.08, .25] | [-.10, .08] | [.15, .32] | [-.11, .07] | [.39, .53] |  |
| 13. EB_all | 2.83 | 0.49 | .31** | .29** | .23** | -.06 | .20** | .46** | .75** | .51** | .73** | .36** | .63** | .45** |
|  |  |  | [.23, .39] | [.21, .38] | [.14, .31] | [-.15, .03] | [.11, .28] | [.39, .53] | [.71, .79] | [.44, .58] | [.69, .77] | [.28, .44] | [.57, .68] | [.38, .52] |

Note: ** Correlation is significant at the 0.01 level. * Correlation is significant at the 0.05 level. RPs_Worry = “How worried are you about climate change?”, RPs_Harm = “How much do you think climate change harms you personally?”, RPs_Urgency = “When do you think climate change will start to harm people in your city?”, RPs_Future = “How much do you think climate change will harm future generations?”, SGS = social generativity scale. Age_group is coded as 0 = younger adults, 1 = older adults. EB_all = mean score of pro-environmental behaviors across all dimensions.

Table 3 Standardized estimates and 95% confidence intervals for linear regression model predicting pro-environmental behaviors, controlling for income, subjective SES, and subjective health

|  | *beta* | *95% CI* | *p* |
| --- | --- | --- | --- |
| AgeGroup (AG) | .24 | [.14, .33] | **<.001** |
| CCRPs_Worry | .16 | [.05, .27] | **.006** |
| CCRPs_Harm | .00 | [-.11, .12] | .937 |
| CCRPs_Urgency | .00 | [-.08, .08] | .917 |
| CCRPs_Future | .04 | [-.06, .14] | .413 |
| SGS | .28 | [.18, .38] | **<.001** |
| Subjective health | .07 | [-.02, .16] | .108 |
| Income | .02 | [-.07, .12] | .619 |
| Sub_SES | .11 | [.03, .20] | **.010** |
| AG × CCRPs_Worry | .00 | [-.11, .12] | .940 |
| AG × CCRPs_Harm | .08 | [-.03, .20] | .141 |
| AG × CCRPs_Urgency | .05 | [-.03, .13] | .216 |
| AG × CCRPs_Future | -.01 | [-.10, .09] | .897 |
| AG × SGS | -.11 | [-.21, -.02] | **.019** |
| R^2^ / R^2^ adjusted | .313 / .291 | | |

Notes: CCRPs = climate change risk perceptions. SGS = social generativity concerns. AG = age groups. SES = socioeconomic status.

Table 4 Unstandardized estimates and 95% confidence intervals for linear regression model predicting pro-environmental behaviors with year as a moderator

| *Predictors* | *beta* | *95% CI* | *p* |
| --- | --- | --- | --- |
| AgeGroup | .21 | [.13, .29] | **<.001** |
| Year | -.12 | [-.19, -.04] | **.004** |
| CCRPs_Worry | .15 | [.04, .26] | **.006** |
| CCRPs_Harm | .01 | [-.10, .12] | .854 |
| CCRPs_Urgency | -.01 | [-.09, .07] | .805 |
| CCRPs_Future | .05 | [-.04, .15] | .290 |
| SGS | .33 | [.24, .42] | **<.001** |
| AgeGroup × Year | -.02 | [-.11, .06] | .615 |
| Year × CCRPs_Worry | .09 | [-.02, .20] | .112 |
| Year × CCRPs_Harm | -.05 | [-.16, .05] | .329 |
| Year × CCRPs_Urgency | .00 | [-.08, .08] | .966 |
| Year × CCRPs_Future | .00 | [-.10, .09] | .956 |
| Year × SGS | -.05 | [-.15, .04] | .238 |
| R^2^ / R^2^ adjusted | .292 / .272 | | |

Notes: ^***^ means p < .001, ^**^ means p < .01, ^*^ means p < .05. CCRPs = climate change risk perceptions. SGS = social generativity concerns. AG = age groups.

Table 5 Standardized estimates and 95% confidence intervals for hierachical regression model predicting pro-environmental behaviors with age groups and reasons

|  | **Pro-environmental: All** | | | | | |
| --- | --- | --- | --- | --- | --- | --- |
| *Predictors* | *beta* | *95% CI* | *p* | *beta* | *95% CI* | *p* |
| R_Environment | .55 | [.43, .67] | **<.001** | .55 | [.41, .69] | **<.001** |
| R_Habit | .20 | [.08, .33] | **.001** | .16 | [.03, .29] | **.015** |
| R_Norm | -.11 | [-.21, -.01] | **.036** | -.08 | [-.18, .03] | .167 |
| R_Money | .07 | [-.04, .17] | .202 | .02 | [-.08, .12] | .705 |
| AG |  |  |  | .15 | [.04, .25] | **.007** |
| AG × R_Environment |  |  |  | .11 | [-.03, .25] | .133 |
| AG × R_Habit |  |  |  | -.01 | [-.15, .12] | .842 |
| AG × R_Norm |  |  |  | .02 | [-.08, .13] | .694 |
| AG × R_Money |  |  |  | .04 | [-.06, .15] | .411 |
| R^2^ / R^2^ adjusted | .482 / .472 | | | .514 / .493 | | |

Notes: R_Environment = pro-environmental reason, R_Habit = habitual reason, R_Norm = norm conformity, R_Money = saving money. AG = age groups, 0 = younger adults, 1 = older adults

Table 6 Standardized estimates and 95% confidence intervals for regression models predicting sub-dimensions of pro-environmental behaviors

|  | **Recycle** | | | **Household** | | | **Consumption** | | |
| --- | --- | --- | --- | --- | --- | --- | --- | --- | --- |
| *Predictors* | *beta* | *95% CI* | *p* | *beta* | *95% CI* | *p* | *beta* | *95% CI* | *p* |
| AG | .58 | [.40, .76] | **<.001** | .01 | [-.19, .20] | .941 | .53 | [.36, .70] | **<.001** |
| CCRPs_Worry | .12 | [-.05, .29] | .164 | .02 | [-.16, .20] | .842 | .02 | [-.14, .18] | .795 |
| CCRPs_Harm | -.13 | [-.30, .05] | .156 | .12 | [-.07, .31] | .220 | -.12 | [-.29, .04] | .141 |
| CCRPs_Urgency | -.04 | [-.16, .09] | .561 | -.09 | [-.22, .05] | .192 | -.07 | [-.19, .05] | .252 |
| CCRPs_Future | .06 | [-.09, .21] | .423 | -.02 | [-.18, .14] | .817 | .14 | [-.00, .28] | .055 |
| SGS | .24 | [.12, .36] | **<.001** | .19 | [.06, .32] | **.005** | .40 | [.28, .51] | **<.001** |
| CCRPs_Worry × AG | -.08 | [-.31, .15] | .483 | .13 | [-.12, .38] | .313 | .11 | [-.11, .33] | .313 |
| CCRPs_Harm × AG | .17 | [-.06, .40] | .154 | -.13 | [-.38, .12] | .317 | .24 | [.02, .46] | **.034** |
| CCRPs_Urgency × AG | .13 | [-.04, .30] | .140 | .10 | [-.09, .28] | .297 | .09 | [-.08, .25] | .301 |
| CCRPs_Future × AG | .11 | [-.10, .31] | .304 | .03 | [-.19, .25] | .777 | -.24 | [-.43, -.05] | **.016** |
| SGS × AG | -.16 | [-.35, .04] | .121 | -.14 | [-.35, .07] | .200 | -.23 | [-.42, -.04] | **.018** |
| R^2^ / R^2^ adjusted | .198 / .179 | | | .059 / .037 | | | .272 / .255 | | |
|  | **Civic activities** | | | **Political participation** | | | **Transportation** | | |
| *Predictors* | *beta* | *95% CI* | *p* | *beta* | *95% CI* | *p* | *beta* | *95% CI* | *p* |
| AG | -.07 | [-.25, .12] | .493 | .31 | [.12, .50] | **.002** | .11 | [-.09 – .31] | .266 |
| CCRPs_Worry | .18 | [.01, .36] | **.043** | .12 | [-.06, .30] | .191 | -.01 | [-.20 – .18] | .921 |
| CCRPs_Harm | -.04 | [-.22, .14] | .679 | .01 | [-.17, .19] | .914 | -.06 | [-.25 – .13] | .532 |
| CCRPs_Urgency | -.03 | [-.16, .10] | .667 | .08 | [-.05, .22] | .206 | .02 | [-.12 – .15] | .807 |
| CCRPs_Future | -.03 | [-.19, .13] | .689 | -.06 | [-.22, .09] | .420 | -.02 | [-.19 – .14] | .776 |
| SGS | .30 | [.17, .43] | **<.001** | .10 | [-.03, .23] | .127 | .08 | [-.05 – .22] | .232 |
| CCRPs_Worry × AG | -.01 | [-.26, .23] | .922 | -.03 | [-.28, .21] | .793 | -.11 | [-.36 – .15] | .419 |
| CCRPs_Harm × AG | .01 | [-.24, .26] | .936 | .08 | [-.17, .32] | .546 | .12 | [-.13 – .38] | .344 |
| CCRPs_Urgency × AG | .12 | [-.06, .30] | .187 | -.09 | [-.27, .09] | .309 | .05 | [-.14 – .24] | .583 |
| CCRPs_Future × AG | .08 | [-.13, .30] | .453 | .03 | [-.19, .24] | .794 | .13 | [-.10 – .35] | .272 |
| SGS × AG | -.19 | [-.39, .02] | .079 | .15 | [-.06, .36] | .166 | -.20 | [-.42 – .01] | .066 |
| R^2^ / R^2^ adjusted | .111 / .089 | | | .101 / .080 | | | .020 / .013 | | |

Notes: CCRPs = climate change risk perceptions. SGS = social generativity concerns. AG = age groups.

Table 7 Group invariance

7.1 One-factor model (overall pro-environmental behaviors):

|  | *df* | AIC | BIC | Chi-square | Chi-square diff | *p* |
| --- | --- | --- | --- | --- | --- | --- |
| Configural | 14 | 6364.7 | 6531.1 | 23.273 |  |  |
| Metric | 19 | 6365.3 | 6510.9 | 33.915 | 10.642 | .060 |
| Scalar | 21 | 6421.8 | 6546.5 | 100.337 | 66.422 | <.001 |
| Scalar (free 3 ints) | 22 | 6365.4 | 6498.5 | 39.974 | 6.0592 | .109 |

Notes: Configural invariance = structural invariance. Metric invariance = factor loadings invariance. Scalar invariance = factor intercepts invariance. Scalar (free 3 ints) model freed the intercepts of the three factors where we found group differences (recycling, consumption, and political support), while keeping the intercepts of the other factors fixed. By doing this, partial scalar invariance was achieved. To provide more unbiased analyses, we used sub-dimensions to compare group mean differences and the overall score for examining associations between variables.

7.2 Six-sub-dimension model

|  | *df* | AIC | BIC | Chi-square | Chi-square diff | *p* |
| --- | --- | --- | --- | --- | --- | --- |
| Configural | 124 | 17263 | 17737 | 174.64 |  |  |
| Metric (fix all) | 132 | 17288 | 17729 | 215.99 | 41.346 | <.001 |
| Metric (fix civic) | 126 | 17292 | 17758 | 208.09 | 33.452 | <.001 |
| Metric (fix recyc) | 126 | 17264 | 17730 | 179.61 | 4.9678 | .083 |
| Metric (fix politic) | 125 | 17262 | 17732 | 175.77 | 1.126 | .288 |
| Metric (fix house) | 125 | 17262 | 17732 | 175.85 | 1.210 | .271 |
| Metric (fix consum) | 126 | 17260 | 17725 | 175.34 | 0.705 | .703 |

Notes: Configural invariance = structural invariance. Metric (fix all) = factor loadings on all six factors were constrained to be the same. Metric (fix civic/recyc/politic/house/comsum) = only factor loadings on civic activities/recycling/political participation/household activities/consumption were constrained. Each model was compared with configural invariance model.

Factor structure (configural) and factor loadings (metric) invariance held for one-factor model. Intercepts (scalar) invariance did not hold for one-factor model, which is reasonable given that older and younger adults scored differently on the overall score of pro-environmental behaviors. Only factor structure invariance held for six-sub-dimension model. We further constrained the factor loadings on each sub-dimension one by one. Older and younger groups loaded only differently on civic activities and invariantly on other sub-dimensions.

Table 8 Standardized estimates and 95% confidence intervals for regression models using continuous age

|  | **Pro-environmental: All** | | |
| --- | --- | --- | --- |
| *Predictors* | *beta* | *95% CI* | *p* |
| Age | 0.19 | 0.11 – 0.28 | **<0.001** |
| CCRPs_Worry | 0.16 | 0.05 – 0.27 | **0.006** |
| CCRPs_Harm | -0.02 | -0.13 – 0.09 | 0.735 |
| CCRPs_Urgency | 0 | -0.08 – 0.08 | 0.999 |
| CCRPs_Future | 0.05 | -0.05 – 0.15 | 0.314 |
| SGS | 0.31 | 0.22 – 0.41 | **<0.001** |
| CCRPs_Worry × AG | 0.01 | -0.10 – 0.13 | 0.801 |
| CCRPs_Harm × AG | 0.08 | -0.04 – 0.20 | 0.17 |
| CCRPs_Urgency × AG | 0.05 | -0.04 – 0.14 | 0.266 |
| CCRPs_Future × AG | -0.01 | -0.11 – 0.09 | 0.87 |
| SGS × AG | -0.11 | -0.20 – -0.01 | **0.023** |
| R^2^ / R^2^ adjusted | 0.275 / 0.257 | | |

Notes: CCRPs = climate change risk perceptions. SGS = social generativity concerns. AG = age groups.

Figure 1 Interaction between continuous age and social generativity on pro-environmental behavior


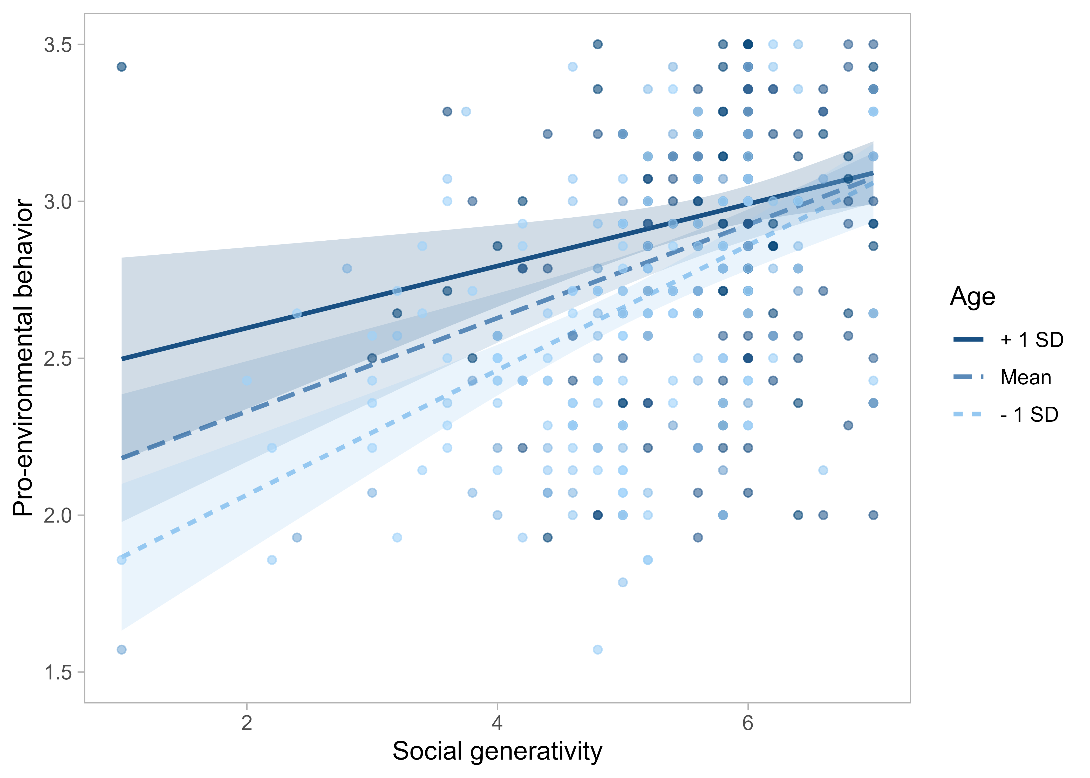


Note: The shadow represents the 95% confidence interval.

Part 9 Deleted items from original pro-environmental scale

| I am an active member of an environmental group |
| --- |
| I systematically take part in protests regarding environmental protection |
| I take part in reforestation or beach cleaning actions |
| I vote a political party that support stronger environmental laws |
| I ride a bicycle or take public transportation to work or school |
| For longer journeys (more than 6 h), I take an airplane/train/bus |
| In winter, I turn down the heat when I leave my apartment for more than 4 h or at night |
| I buy seasonal produce |
